# *Shigella* with Decreased Susceptibility to Azithromycin Among Men Who Have Sex with Men — United States, 2002–2013

**Published:** 2014-02-14

**Authors:** Katherine E. Heiman, Maria Karlsson, Julian Grass, Becca Howie, Robert D. Kirkcaldy, Barbara Mahon, John T. Brooks, Anna Bowen

**Affiliations:** 1 Division of Foodborne, Waterborne, and Environmental Diseases, National Center for Emerging and Zoonotic Infectious Diseases, CDC; 2 Division of STD Prevention, National Center for HIV, Hepatitis, STD, and TB Prevention, CDC; 3 Division of HIV/AIDS Prevention, National Center for HIV, Hepatitis, STD, and TB Prevention, CDC

Bacteria of the genus *Shigella* cause approximately 500,000 illnesses each year in the United States. Diarrhea (sometimes bloody), fever, and stomach cramps typically start 1–2 days after exposure and usually resolve in 5–7 days.* For patients with severe disease, bloody diarrhea, or compromised immune systems, antibiotic treatment is recommended, but resistance to traditional first-line antibiotics (e.g., ampicillin and trimethoprim-sulfamethoxazole) is common. For multidrug-resistant cases, azithromycin, the most frequently prescribed antibiotic in the United States (1), is recommended for both children and adults (2,3). However, not all *Shigellae* are susceptible to azithromycin (4–6). Nonsusceptible isolates exist but are not usually identified because there are no clinical laboratory guidelines for azithromycin susceptibility testing. However, to monitor susceptibility of *Shigellae* in the United States, CDC’s National Antimicrobial Resistance Monitoring System (NARMS) has, since 2011, routinely measured the azithromycin minimum inhibitory concentration (MIC) for every 20th *Shigella* isolate submitted from public health laboratories to CDC, as well as outbreak-associated isolates. All known U.S. *Shigella* isolates with decreased susceptibility to azithromycin (DSA-*Shigella*), and the illnesses caused by them, are described in this report.

DSA-*Shigella* is defined as a *Shigella* isolate with an azithromycin MIC >16 *μ*g/mL (4). Twenty-nine DSA-*Shigella* isolates were identified through routine NARMS testing. Additional isolates from 2002–2013 were identified through a previous NARMS study (n = 3) (4), requests to public health officials (n = 2), and retrospective testing of available isolates with pulsed-field gel electrophoresis (PFGE) patterns indistinguishable from DSA-*Shigella* isolates (n = 21).

Among 55 patients from 17 states infected with DSA-*Shigella* (36 *S. flexneri*, 18 *S. sonnei*, one *S. boydii*), age ranged from 1 to 89 years (median: 42 years); 44 (80%) were men, and seven (13%) were children (aged <18 years). Of 35 patients for whom information was available, 23 (66%) were white, 11 (31%) were black, and one (3%) was Asian/Pacific Islander (two patients self-identified as white and Hispanic and one as Hispanic only). All but one patient resided in an urban area; one child and none of 29 adults for whom information was available reported international travel. Four patients were part of a recognized shigellosis outbreak (5). The median duration of illness was 11 days (n = 17). Of patients for whom information was available, 46% (12 of 26) had bloody diarrhea, 50% (16 of 32) had fever, and 45% (19 of 42) were hospitalized. Eighty-one percent (13 of 16) of men for whom information was available were human immunodeficiency virus (HIV)–positive, and 79% (11 of 14) identified as gay, bisexual, or other men who have sex with men (collectively referred to as MSM). Four men reported recent high-risk sexual practices, including anonymous sexual contact (n = 1), sexual contact without a barrier (n = 2 anal-genital; n = 1 oral-anal), and many sexual partners (n = 1); five had a history of syphilis.

All isolates harbored *mph*A or *erm*B macrolide resistance genes that are commonly plasmid-encoded. Fifty-three percent (29 of 55) were resistant to five or more classes of antibiotics, and 4% (2 of 55) were resistant to ciprofloxacin. NARMS data indicated that isolates were not susceptible to the drug used for treatment in seven of 19 patients, including three treated with azithromycin.

DSA-*Shigella* infections are occurring in the United States. Although some of the infections occurred among children, who are often treated with azithromycin for shigellosis, these data suggest that MSM, especially HIV-infected MSM, are currently at greater risk for infection with DSA-*Shigella*. Shigellosis is more common and can be more severe among HIV-infected persons with CD4 cell counts <200/mm^3^ (7). Clinical failure of azithromycin was recently reported in a Dutch HIV-infected patient with shigellosis (6). Clinicians should be aware that MSM and HIV-positive persons with shigellosis might be infected with *Shigella* strains with reduced susceptibility to azithromycin. Clinicians should culture stool specimens of MSM and HIV-infected men experiencing diarrhea and determine antimicrobial susceptibility of *Shigella* to antibiotics other than azithromycin to help guide treatment, if needed. Meticulous handwashing and reducing fecal-oral exposures during sexual contact can reduce risk for infection (7).

The number of cases presented in this report is likely a substantial underestimate because NARMS routinely tests only 5% of *Shigella* isolates submitted to public health laboratories, and targeted testing using PFGE might miss cases because *Shigella* is highly mutable and plasmid-encoded macrolide resistance genes are mobile. Additionally, because NARMS began routinely measuring susceptibility to azithromycin in 2011, and recent isolates were more likely to be available for retrospective analysis, these data provide no information about trends. To better track illnesses and guide patient management, clinical laboratory guidelines for azithromycin susceptibility testing among *Enterobacteriaceae* are urgently needed.

## Footnotes

* Additional information available at http://www.cdc.gov/nczved/divisions/dfbmd/diseases/shigellosis.
